# The Book World of Medicine and Science

**Published:** 1903-04-18

**Authors:** 


### The Book World of Medicine and Science.

Aids to Gynæcology. By Alfred S. Gubb, M.D. Paris, M.R.C.S. Eng. Fourth edition. (London: Baillière, Tindall and Cox. 1903. Price 2s. 6d. cloth, 2s. paper.)

This edition of Dr. Gubb's useful little book is a great advance on those which have gone before. It has been almost entirely rewritten and in many ways brought up to date. Various illustrations also have been added to elucidate the meaning of the text. That gynæcology has grown into far too large a subject to be capable of comprehensive treatment in a little book of 135 pages goes without saying, but as a "grind book" by aid of which to run over knowledge previously obtained from other sources, or as a means of putting in orderly form the more or less disconnected facts observed in the wards or the out-patient department, this little handbook will, we think, serve a very useful purpose. Throughout the book much trouble is taken to set out the matter in systematic form under carefully numbered headings and sub-headings, all of which, while very useful in its own way, sometimes leads to curious results, such, for example, as the consideration of all kinds of hæmorrhage from the genitals under the heading of "Disorders of Menstruation." As showing how persistently ancient methods retain their place in text-books, we note that in describing ovariotomy Dr. Gubb still refers to the use of the clamp as a means of securing the pedicle. A curious error appears on page 78, where a couple of illustrations are given showing "modes of rupture of intra-uterine gestation sac." Of course, *extra*-uterine is what is meant, gestation being in each case represented as occurring in the Fallopian tubes. We think that the book will be found much more useful as an aid to study than as a guide to practice — which probably is what was intended by the author.

The Errors of Accommodation and Refraction of the Eye and their Treatment. By Ernest Clarke, F.R.C.S., M.D., etc. (Small 8vo., pp. 213, with numerous illustrations. Baillière, Tindall and Cox. 1903.)

Dr. Clarke describes his work as a "handbook for students," and tells us, in a preface which has the merit of brevity, that he has "omitted all matter unnecessary for the busy practitioner or overburdened student." We should ourselves describe it as an industrious compilation from many sources, the list of which, given as a "bibliography," might be largely extended, while the gathering together has not been guided on all points by any considerable degree of practical knowledge of the questions at issue, or by allotments of space bearing any discoverable proportion to their relative importance. It is well known, for example, that a very great man, Benjamin Franklin, first suggested the use of divided lenses, to be worn by persons who require one power for distance and another for near work; and that frames so fitted are properly called "Franklin spectacles." Such spectacles are of the greatest possible comfort and utility to large numbers of people; but Dr. Clarke devotes only 16 lines to them, which are divided between two separate parts of his book, and which, even in their brief compass, contain serious practical errors. He says, for instance, that a patient wearing such spectacles "should be warned to lift the glasses when going up and down steps and stairs." This necessity can only arise when the line of division between the two half-lenses is placed too high; and somewhere in the book we observed a recommendation, not noted in the index, that this line should be level with the lower margin of the iris, which is much too high, and fully explains the "steps and stairs" recommendation. The line of division should always be well below the margin of the lower lid; and, when it is so placed, "steps and stairs" present no difficulties. A still more important error is to say that "the reading part may be cemented on with balsam." This "may" be done, of course, and possibly might be done by any person who would follow Dr. Clarke's example by giving the result a name in shopkeeper's Greek, and by speaking of "pantoscopic," instead of "Franklin" spectacles. An essential characteristic of the latter is that each ring of the frame carries two separate half-lenses, the upper ones occupying a vertical position before the eyes, while the lower are in a plane which forms an obtuse angle with the former in such a way that, whether the eyes are directed forwards at distant objects, or downwards at a book, the lines of sight are always normal to the surfaces of the lenses actually in use. If flat glasses of double focus are placed before the eyes, the surfaces of the glasses must be oblique to the line of sight either when looking forwards or when looking downwards; and it is unnecessary to say that a lens placed obliquely distorts everything that is seen through it, and does so in proportion to its strength. Franklin glasses are chiefly worn by hypermetropic presbyopes, who, as a rule, require strong reading glasses; and hence, if they were to receive vertically-placed cemented lenses they would be seriously embarrassed by the faulty position of the lower and stronger parts in relation to the line of sight in reading.

Another trifling indication of the general character of the book is furnished by the circumstance that Dr. Clarke, in speaking of the means of detecting astigmatism, mentions and figures something which he calls "Brudenell Carter's clock," but which does not bear the smallest resemblance, either in appearance or mode of action, to the "dial" which Mr. Carter contrived and described many years ago. There are the figures of a clock-face on both, just as there is a river in Macedon and a river in Monmouth, but that is all that can be said.

### BOOKS RECEIVED.

Longmans, Green and Co.

"Colonial and Camp Sanitation." By G. V. Poore, M.D.

Gee and Co.

"Medical Practitioners' Accounts." By J. H. May.

The General Medical Council.

"The Dentist's Register, 1903."
"The Medical Register, 1903."

Baillière, Tindall and Cox.

